# Erythrocyte and spermatozoa glucose-6-phosphate dehydrogenase activity in merino rams: An experimental study

**Published:** 2018-06

**Authors:** Hüseyin Gurel, Nuri Baspinar, Pınar Peker Akalin, Vahdettin Altunok, Filiz Kazak

**Affiliations:** 1 *Lütfü Kırdar Education and Training Hospital, Istanbul, Turkey.*; 2 *Department of Biochemistry, Veterinary Faculty, Selcuk University, Konya, Turkey.*; 3 *Department of Biochemistry, Veterinary Faculty, Mustafa Kemal University, Hatay, Turkey.*

**Keywords:** Spermatozoa, Erythrocyte, Ram, Glucose-6-phosphate dehydrogenase

## Abstract

**Background:**

Glucose-6-phosphate dehydrogenase (G6PD) is the first enzyme of the pentose phosphate metabolic pathway that supplies reducing agents by maintaining the level of reduced nicotinamide adenine dinucleotide phosphate.

**Objective:**

It was aimed to determine the activity of erythrocyte and spermatozoa G6PD in the breeding and non-breeding seasons in Merino rams. And also, to find out the relation of these parameters with sperm quality parameters for better understanding the role of this enzyme in male fertility.

**Materials and Methods:**

1.5-2 yr-old healthy, 14 Merino rams were involved. Ejaculate samples were collected using an artificial vagina, in October (the breeding season) and April (the non-breeding season). Blood samples were collected prior to sperm collection. Sperm volume (ml), motility (%), mass activity (1-5), concentration (×10^6^), viability (%), abnormal acrosome morphology (%) and abnormal sperm morphology (%) was evaluated. The activities of spermatozoa and erythrocyte G6PD were determined and the relation of sperm parameters with G6PD activity was evaluated.

**Results:**

Erythrocyte G6PD activity was higher (p≤0.001), whereas spermatozoa G6PD activity was lower (p≤0.001) in the breeding season (1.928±0.231 U/g hemoglobin, 129.65±28.41 U/g protein, respectively) from that in the non-breeding (0.530±0.066 U/g hemoglobin, 562.36±94.92 U/g protein, respectively). There were also significant differences among sperm quality parameters within the seasons. Positive correlation was determined between spermatozoa G6PD activity (r=0.053, p=0.03 and sperm concentration in the breeding season.

**Conclusion:**

Higher spermatozoa G6PD activity in October, where the level of polyunsaturated fatty acids is suggested to be increased, may reflect the increased need of nicotinamide adenine dinucleotide phosphate and thus higher G6PD activity for the oxidative balance.

## Introduction

Glucose-6-phosphate dehydrogenase (D-glucose 6-phosphate: NADP+ oxidoreductase EC 1.1.1.49; G6PD) regulates the pentose phosphate metabolic pathway that supplies reducing agents by maintaining the level of reduced nicotinamide adenine dinucleotide phosphate (NADPH). Therefore, it is a regulator of redox balance (1). It is reported that, ram sperm have higher polyunsaturated fatty acids than other species, in their sperm membrane, which increase vulnerability of the sperm to oxidative damage caused by free radicals such as reactive oxygen species (ROS). Cell functions, that are sperm motility, fertilizing capability and morphology, are negatively affected by the production of excess ROS (2, 3). In spermatozoa, glucose is metabolized by glycolysis and the pentose phosphate pathway (4, 5). Luna and colleagues,reported that in ram sperm, G6PD is localized at the postacrosomal region and tail, especially in the midpiece of fresh spermatozoa (6). G6PD activity varies according to the stage of development of sperm cells; its activity is the highest in spermatocytes, lower in the spermatids and the lowest in spermatozoa (7). Semen production is influenced by climatic factors such as daylight time, temperature and humidity, in rams (8). Seasonal changes of seminal plasma antioxidant enzyme activities (Glutathione reductase, glutathione peroxidase, catalase and superoxide dismutase) have been reported in Rasa Aragonesa rams (9). 

The relationship between human erythrocyte and spermatozoa G6PD activity was reported (10). The activity and mRNA levels of semen G6PD was investigated in men, living in highly polluted areas (11). After environmental exposure to lead, human spermatozoa glutathione reductase activity was reduced by 47% (p<0.05), where catalase was increased by 109% (p<0.05) and G6PD was increased by 37% (p>0.05) (12). Sperm G6PD activity was reported to be lower in G6PD deficient men than those in normal individuals (10), on the other hand, G6PD deficiency seemed not to increase the susceptibility of human sperm to oxidative stress induced by H_2_O_2_ (13). G6PD activity was lower in Beagle dogs with asthenozoospermia, compared to healthy ones. Also, sperm volume, concentration and motility were positively correlated with G6PD activity (14). 

To our knowledge, the seasonal variation of G6PD activity, both in erythrocyte and spermatozoa, was not reported in rams. Also, the relationship between G6PD activity and sperm quality parameters was not reported. As ram semen is more vulnerable to oxidative damage than other species (2, 3).

We aimed to investigate erythrocyte and spermatozoa G6PD activities and the relationship with sperm quality parameters in Merino rams in the breeding and the non-breeding seasons, to better understand the role of this antioxidant enzyme on ram semen.

## Materials and methods

**Material**

Semen and blood (erythrocyte) samples from 14 healthy Merino Rams (1.5-2 yr) were involved in the study. The rams were maintained under uniform feeding and housing (located at 37.857063 north latitude and 32.567036 east longitude) conditions and were fed with a ration composed of alfalfa hay, concentrated feed, corn silage and dried grape, had ad libitum access to fresh water. Group feeding was accessed. 

**Methods**

Ejaculate samples were collected from the rams using an artificial vagina, in October (the breeding season) and April (the non-breeding season) according to artificial insemination standard procedures (15).

**Evaluation of sperm parameters**

Immediately after collection, the volume of ejaculates was evaluated in a conical tube graduated at 0.1 mL intervals (16). Using a phase-contrast microscope, semen mass activity was assessed at 40×magnification, graded on a subjective scale ranging from 1-5, where 1 represented no mass movement and 5 represented vigorous waves of sperm motion (16).

Semen was diluted with PBS (Sigma P4417) (1/10 w/w) and a wet mount was made using a 5 µL drop of this dilution, placed directly on a microscope slide for determining subjective sperm motility (by a phase-contrast microscope (100×)). Motility estimations were performed in three different microscopic fields for each semen sample by the same researcher and the mean of the three successive estimations was recorded (16).

Sperm concentration was determined via hemositometric method. Briefly, sperm was diluted at ratio of 1:200 with Hayem solution (5 g Na_2_SO_4_ (Sigma 239313), 1 g NaCl (Sigma S9888), 0.5 g HgCl_2_ (Sigma V001972), 200 ml bicine (Aldrich 163791) and density was determined using a 100 μm deep Thoma haemocytometer (TH-100, Hecht-Assistent, Sondheim, Germany) at 400× magnification by using a phase-contrast microscope and expressed as spermatozoa × 10^9^ ml−1 (17). 

Hypo-osmotic swelling test was performed to determine the functional integrity of the sperm membrane (Viability), by incubating 30 µL of semen with 300 µL of a 100 mOsm hypoosmotic solution (4.9 g sodium citrate+9 g fructose for a liter of distilled water) at 37^o^C for 60 min. 400 sperms were evaluated for each sample, and the percentage of spermatozoa with swollen and twisted tails were recorded under phase-contrast microscope (400×) (18). 

To evaluate sperm morphology, a minimum of three drops of each sample were added to tubes containing 1 ml Hancock solution (150 ml of saline, 62.5 ml of formalin (37%) (Sigma F8775), 150 ml of buffer solution and 500 ml of distilled water). The percentage of total abnormal sperm morphology (abnormal acrosomal morphology+other abnormal sperm morphology) was recorded by counting a total of 400 sperm cells under phase-contrast microscopy (1000× magnification) (19).

**Determination of erythrocyte and spermatozoa G6PD activities**

Blood, collected in EDTA (Sigma-E1644), prior to sperm collection, was centrifuged at 600g ×10 min × +4^o^C. The red blood cells were isolated and washed three times with 0.16 M KCl (Sigma P9333). Erythrocyte pellet was stored at -86oC until the analysis of the enzyme. Just before the analysis of the enzyme, erythrocyte pellet was suspended with ice-cold distilled water and the hemolysate was centrifuged at 13000 g × 15 min × +4^o^C, supernatant was used for the enzyme analysis.

Ejaculates were centrifuged at 600g ×10 min +4^o^C and seminal plasma was removed. The cellular pellet was washed three times with 0.16 M KCl. Cellular pellet was resuspended in 1 ml 0.16 M KCl and stored at -86^o^C until the analysis of the enzyme. Just before the analysis of the enzyme, 1 ml spermatozoa in 0.16 M KCl was sonicated in 2 ml transparent, polyethylene tubes (1.5 cm in diameter, 6 cm in height) with continuous basis, by SONIC vibra cells (Sonics & Materials, INC, USA, model: VCX 130, Serial no: 45822, net power output 130 W, Frequency 20 kHz, Amplitude 100%, Prob: S&M 630-0422, Prob Model: CV18, Prob Serial No: 6837) for 5 repretitive and 10 sec duration with 30 sec cooling period (in ice) between each duration time. Then the homogenate was centrifuged at 13000 g × 15 min x +4^o^C and supernatant was used for the enzyme analysis (20).

G6PD activity was measured as described by Beutler (21), spectrophotometrically. Briefly, the enzyme sample was added to the 1 ml of (final volume) incubation mixture containing 100 µl 1 M Tris(VWR 28811.295)-HCl (Sigma H1758)-EDTA (pH 8.0)+100 µl 0,1 mol/L MgCl_2 _(Sigma M2670), 100 µl 2 mM NADP+(Sigma N8160) and 100 µl 6 mM glucose-6 phosphate (Sigma G7879), distilled water and the supernatant. The increase in absorption at 340 nm due to the reduction of NADP^+^ at 25^o^C was recorded. 

**Determination of protein and hemoglobin levels**

Total protein levels was determined by Bradford Method, using bovine serum albumin as standard (22). Hemoglobin levels were determined with Drabkin’s (Sigma Katalog No: D5941-6 VL) solution.

**Ethical consideration**

This study was approved by Selcuk University Veterinary Faculty Local Animal Research Ethics Committee (No: 2011/007) and the procedures have been approved and also care was taken to minimize the number of animals used.

**Statistical analysis**

Statistical analyses were performed with the SPSS (Statistical Package for the Social Sciences, version 12.00, Chicago, IL, USA) program. Results were expressed as the mean±SEM. Means between the seasons were analyzed by Students t-test. Correlation between erythrocyte and spermatozoa G6PD activities and sperm parameters were performed with Pearson correlation and considered significant at p<0.05.

## Results

Erythrocyte and spermatozoa G6PD activities in October (Breeding) and April (Non-breeding) and the correlation between sperm parameters are shown in table I and II. Erythrocyte G6PD activity was higher (p≤0.001) in the breeding season (1.928±0.231 U/g hemoglobin) from that in the non-breeding season (0.530±0.066 U/g hemoglobin). Spermatozoa G6PD activity was lower (p≤0.001) in the breeding season (129.65±28.41 U/g protein) compared to the non-breeding season (562.36±94.92 U/g protein). Spermatozoa total protein levels were found to be lower (p=0.004) in the breeding season (7.88±2.24 mg/dl) compared to the non-breeding season (14.99±5.38 g/dl). Among the analyzed sperm parameters, volume of ejaculates (p=0.004), viability (p=0.026) and abnormal acrosome morphology rate (p=0.006) were higher in the breeding season (2.36±0.16 ml, 82.93±3.49%, 2.71±0.27%, respectively) than in the non-breeding season (1.76±0.12 ml, 73.21±2.32%, 1.29±0.34%, respectively). Abnormal sperm morphology rate was lower in the breeding season (7.36±0.68%, p=0.000) compared to the non-breeding season (18.21±0.87%) (Table I).

As regards correlation values, there was only a positive correlation between concentration and spermatozoa G6PD activity (p=0.03) in October (Table II).

**Table I:** Erythrocyte and spermatozoa G6PD activities and some sperm parameters (Mean±SE, n=14) in October and April in merino rams

	**October (breeding)**	**April (non-breeding)**	**p-value**
Erythrocyte G6PD (U/g hemoglobin)	1.928 ± 0.231^a^	0.530 ± 0.066 b	0.000
Spermatozoa G6PD (U/g protein)	129.65 ± 28.41^a^	562.36 ± 94.92 b	0.000
Spermatozoa total protein (g/dl)	7.88 ± 0.22 a	14.99 ± 0.54 b	0.000
Volume of ejaculates (ml)	2.36 ± 0.16^a^	1.76 ± 0.12^b^	0.004
Mass activity (1-5)	3.07 ± 0.07	3.14 ± 0.18	-
Subjective Motility (%)	83.57 ± 1.33	83.57 ± 1.10	-
Concentration (×10^6^)	2330.36 ± 198.00	2611.43 ± 127.79	-
Viability (%)	82.93 ± 3.49^a^	73.21 ± 2.32^b^	0.026
Abnormal acrosome morphology rate %	2.71 ± 0.27^a^	1.29 ± 0.34^b^	0.006
Abnormal sperm morphology rate %	7.36 ± 0.68^a^	18.21 ± 0.87^b^	0.000

Data presented as mean±SD.

G6PD: Glucose-6-phosphate dehydrogenase activity.

a, b : The same letters in the same row are statistically significant.

**Table II:** Correlations (r values) of erythrocyte and spermatozoa G6PD activities with sperm parameters in merino rams (n=14)

	**October (breeding)**		**April (non-breeding)**	
	**Erythrocyte G6PD (U/g hemoglobin)**	**Spermatozoa G6PD (U/g protein)**	**Erythrocyte G6PD (U/g hemoglobin)**	**Spermatozoa G6PD (U/g protein)**
Sperm G6PD (U/g protein)	0.013	-	-0.104	-
Volume of ejaculates (ml)	0.362	0.440	-0.255	-0.197
Mass activity (1-5)	-0.066	-0.291	-0.158	-0.314
Subjective motility (%)	0.008	-0.178	-0.213	-0.047
Concentration (x106)	-0.115	0.553*	0.418	-0.105
Viability (%)	-0.173	0.129	0.363	-0.154
Abnormal acrosome morphology rate %	0.338	0.052	-0.511	-0.001
Abnormal sperm morphology rate %	-0.444	-0.114	0.029	0.514

G6PD: Glucose-6-phosphate dehydrogenase activity.

* p<0.03

## Discussion

G6PD is the first enzyme of the pentose phosphate metabolic pathway that supplies reducing agents by maintaining the level of the co-enzyme NADPH. It reduces nicotinamide adenine dinucleotide phosphate (NADP^+^) to NADPH, while oxidizing glucose-6-phosphate (23). The NADPH in turn, maintains the level of reduced glutathione (GSH) in the cell which protects red blood cells against oxidative damage (24).

In this study, erythrocyte G6PD activities in Merino rams in October, were in accordance with the literature, whereas, the levels were significantly lower in April. Sheep have the lowest G6PD activity in their erythrocyte, among the animals. It was reported that the activities are 9.44±1.18; 13.82±1.19; 11.0±2.70; 11.86±1.52; 10.6±3.79; 1.98±0.27 and 1.20±0.37 U/g Hb in the guinea pig, rat, dog, rabbit, monkey, goat and sheep, respectively (25). Erythrocyte G6PD activity also varies among sex characters; 1.81 U/g Hb in Rahmani rams and 1.14 U/g Hb in Rahmani sheep (26). To our knowledge, this is the first study reporting the activity of erythrocyte G6PD in different seasons. The difference between October and April is suggested to be caused by seasonal changes such as daylight time, temperature and humidity.

In this study, spermatozoa G6PD activity was determined in the spermatozoa supernatant and based on protein levels (Between 129-562 µmol/NADPH/min/g protein, U/g protein). G6PD activity was reported to be between 0.01-0.02 nmol/NADPH/10^6^ spermatozoa, in Rasa Aragonesa rams (6). In the literature (14), based on protein levels, G6PD activity of healthy Beagle dog sperm was reported to be 11.3±3.0 nmol/min/mg protein (11.3±3.0 U/g protein). 

Semen production is influenced by climatic factors such as daylight time, temperature and humidity, in rams (27, 28). Marti and colleagues (9) reported that antioxidant enzymes changed throughout the year, in Rasa Aragonesa rams. Glutathione reductase, glutathione peroxidase, catalase and superoxide dismutase activities were 3.12±0.31, 6.85±0.78, 8.73±0.77, 1.02±0.06 nmol/min/mg protein in the breeding season, respectively, whereas their activities were 4.14±0.22, 6.91±0.69, 16.70±1.55, 1.44±0.08 nmol/min/mg protein in the non-breeding season, respectively. Catalase activity was higher in the non-breeding season. The higher levels of spermatozoa G6PD activity in the non-breeding season (April) is in accordance with Marti colleagues, who reported increased levels of seminal plasma antioxidant enzymes in the non-breeding season (9). The increased need of antioxidant potential and NADPH in the non-breeding season may be compensated by increased G6PD activity. The higher levels of antioxidant enzymes in ram seminal plasma in the non-breeding season and higher G6PD activity in spermatozoa in the non-breeding season in the current study can be attributed to the protective effects of G6PD against increased levels of ROS (9). Thus, in summer season, poly-unsaturated fatty acid (PUFA) levels in ram spermatozoa increase. Docohexaenoic acid (C22:6n³) levels were reported to be %24, 94±0.74 in winter and 30.05±0.74% in summer, and arachidonic acid (C20: 4n6) levels were 0.97±0.05% in winter and 1.23±0.03% in summer (9). As the levels PUFA increase in spermatozoa and seminal plasma, the ROS levels increase and the vulnerability of the sperm membrane to ROS increases (29, 30). 

Thus, Yeni and colleagues, reported higher seminal plasma MDA levels in summer and autumn compared to winter in rams (31). Enzymatic and non-enzymatic antioxidants protect the sperm membrane from lipid peroxidation (9). Luna and colleagues also stated that there was relationship between the production of NADPH via the pentose phosphate pathway and sperm capacitation and fertilization (6). The antioxidant effects of G6PD may be attributed to its relation with fertility. Also, the higher levels of total protein in the non-breeding season in the current study may reflect the increased levels of antioxidant enzymes, needed for the elimination of excess ROS. Overall, the relationship of G6PD with different seasons needs further studies in order to investigate its role in fertility of rams.

## Conclusion

In conclusion, erythrocyte G6PD activity decreased and spermatozoa G6PD activity increased in October compared to April. A positive correlation was determined between spermatozoa G6PD activity and sperm concentration, in October. Higher spermatozoa G6PD activity in October, where the levels of PUFA is suggested to be increased, may reflect the increased need of NADPH and thus higher G6PD activity for the oxidative balance.

## Note

***This article extracted from M.Sc. Thesis. (Huseyin Gurel)***

## References

[1] Ho HY, Cheng ML, Chiu DT (2007). Glucose-6-phosphate dehydrogenase-from oxidative stress to cellular functions and degenerative diseases. Redox Rep.

[2] Aitken J, Fischer H (1994). Reactive oxygen species generation and human spermatozoa: the balance of benefit and risk. Bioassays.

[3] Gadea J, Selles E, Marco MA, Coy P, Matas C, Romar R (2004). Decrease in glutathione content in boar sperm after cryopreservation, Effect of the addition of reduced glutathione to the freezing and thawing extenders. Theriogenology.

[4] Urner F, Sakkas D (1999). A possible role of the pentose phosphate pathway of spermatozoa in gamete fusion in the mouse. Biol Reprod.

[5] Miraglia E, Lussiana C, Viarisio D, Racca C, Cipriani A, Gazzano E (2010). The pentose phosphate pathway plays an essential role in supporting human sperm capacitation. Fertil Steril.

[6] Luna C, Serrano E, Domingo J, Casao A, Pérez-Pé R, Cebrián-Pérez JA (2016). Expression, cellular localization, and involvement of the pentose phosphate pathway enzymes in the regulation of ram sperm capacitation. Theriogenology.

[7] Bajpai M, Gopal G, Setty BS (1998). Changes in carbohydrate metabolism of testicular germ cells during meiosis in the rat. Eur J Endocrin.

[8] Azawi OI, Ismaeel MA (2012). Effects of seasons on some semen parameters and bacterial contamination of Awassi ram semen. Reprod Domest Anim.

[9] Marti E, Mara L, Marti JI, Muino-Blanco T, Cebrian-Perez JA (2007). Seasonal variations in antioxidant enzyme activity in ram seminal plasma. Theriogenology.

[10] Sarkar S, Nelson AJ, Jones OW (1977). Glucose-6-phosphate dehydrogenase activity of human sperm. J Med Genet.

[11] Bergamo P, Volpea MG, SLorenzetti S, Mantovani A, Notari T, Cocca E (2016). Human semen as an early, sensitive biomarker of highly pollutedliving environment in healthy men: A pilot biomonitoring study ontrace elements in blood and semen and their relationship with spermquality and Redox status. Reprod Toxicol.

[12] Kasperczyk A, Dobrakowski M, Czuba ZP, Horak S, Kasperczyk S (2015). Environmental exposure to lead induces oxidative stress and modulates the function of the antioxidant defense system and the immune system in the semen of males with normal semen profile. Toxicol Appl Pharmacol.

[13] Roshankhah S, Rostami-Far Z, Shaveisi-Zadeh F, Movafagh A, Bakhtiari M, Shaveisi-Zadeh J (2016). Glucose-6-phosphate dehydrogenase deficiency does not increase the susceptibility of sperm to oxidative stress induced by H2O2. Clin Exp Reprod Med.

[14] Kawakami E, Arai T, Nakamura U (1999). Effects of medium containing heparin and theophylline on capacitation and metabolic enzyme activities of ejaculated spermatozoa from dogs with asthenozoospermia. Anim Reprod Sci.

[15] Paulenz H, Söderquist L, Perez-Pe R, Berg KA (2002). Effect of different extenders and storage temperatures on sperm viability of liquid ram semen. Theriogenology.

[16] Evans G, Maxwell WMC, Maxwell W.M.C (1987). Handling and Examination of Semen. Salamon’s Artificial Insemination of Sheep and Goat.

[17] Bearden HJ, Fuquay JW, Willard ST ( 2004). Applied Animal Reproduction.

[18] Revel SG, Mrode RA (1994). An osmotic resistance test for bovine semen. Anim Reprod Sci.

[19] Schafer S, Holzmann A (2000). The use of transmigration and sperma stain to evaluate epididymal cat spermatozoa. Anim Reprod Sci.

[20] Akalın PP, Başpinar N, Çoyan K, Bucak MN, Güngör Ş, Öztürk C (2016). Effects of ultrasonication on damaged spermatozoa and mitochondrial activity rate. Turk J Vet Anim Sci.

[21] Beutler E (1984). Red cell metabolism. A manuel of Biochemical Method’s.

[22] Bradford MM (1976). A rapid and sensitive method for the quantitation of microgram quantitation of protein utilizing the principle of protein-dye binding. Anal Biochem.

[23] Kletzien RF, Harris PK, Foellmi LA (1994). Glucose-6-phosphate dehydrogenase: a "housekeeping" enzyme subject to tissue-specific regulation by hormones, nutrients, and oxidant stress. FASEB J.

[24] Salati LM, Amir-Ahmadi B (2001). Dietary regulation of expression of glucose-6-phosphate dehydrogenase. Annu Rev Nutr.

[25] Cheun LH (1966). Glucose-6-phosphate dehydrogenase activity in erythrocytes of experimental animals. J Clin Pathol.

[26] Maronpot RR (1972). Erythrocyte glucose-6-phosphate dehydrogenase and glutathione deficiency in sheep. Can J Comp Med.

[27] Yoshida A, Stamatoyannopoulos G, Motulsky AG (1967). Negro variant of glucose-6-phosphate dehydrogenase deficiency (A-) in man. Science.

[28] Folch J, Courot M, Martinus N (1984). Influence of age, photoperiodism and temperature on semen production of rams. The Male In Farm Animal Reproduction.

[29] Argov-Argaman N, Mahgrefteh K, Zeron Y, Roth Z (2013). Season-induced variation in lipid composition is associated with semen quality in Holstein bulls. Reproduction.

[30] Alvarez JG, Storey BT (1995). Differential incorporation of fatty acids into and peroxidative loss of fatty acids from phospholipids of human spermatozoa. Mol Reprod Dev.

[31] Yeni D, Gündoğan M, Ciğerci İH, Avdatek F, Fidan AF (2010). Seasonal variation of oxidative stress parameters in ram seminal plasma. J Anim Vet Adv.

